# Cholesterol induces lipoprotein lipase expression in a tree shrew (*Tupaia belangeri chinensis*) model of non-alcoholic fatty liver disease

**DOI:** 10.1038/srep15970

**Published:** 2015-11-02

**Authors:** Linqiang Zhang, Zhiguo Zhang, Yunhai Li, Shasha Liao, Xiaoyun Wu, Qing Chang, Bin Liang

**Affiliations:** 1 Key Laboratory of Animal Models and Human Disease Mechanisms of Chinese Academy of Science & Yunnan province, Kunming Institute of Zoology, Chinese Academy of Sciences, Kunming, Yunnan 650223, China; 2 Kunming College of Life Science, University of Chinese Academy of Sciences, Kunming, Yunnan 650204, China; 3 School of Life Sciences, Anhui University, Hefei, Anhui 230601, China

## Abstract

Animal models are indispensible to investigate the pathogenesis and treatments of non-alcoholic fatty liver diseases (NAFLD). Altered cholesterol metabolism has been implicated into the pathogenesis of NAFLD. Here, using high fat, cholesterol and cholate diet (HFHC), we generated a novel tree shrew (*Tupaia belangeri chinensis*) model of NAFLD, which displayed dyslipidemia with increased levels of plasma alanine aminotransferase (ALT) and aspartate aminotransferase (AST), total cholesterol (TC), low density lipoprotein-cholesterol (LDL-c) and high density lipoprotein-cholesterol (HDL-c), but decreased level of triglycerides (TG). Liver histopathology and genes expression indicated that HFHC diet successfully induced liver steatosis to inflammation and fibrosis progressively within 10 weeks. Moreover, HFHC induced the transcriptional expression of lipoprotein lipase (*lpl*) in the liver, but repressed the expression of LDL receptor, and the endogenous synthesis pathway and excretion of cholesterol. Notably, Poloxamer 407 (P-407) inhibition of LPL improved the severity of steatosis and reduced inflammation. These results illustrated that LPL plays an important role in cholesterol metabolism in NAFLD, and the tree shrew may be a valuable animal model for further research into NAFLD.

Non-alcoholic fatty liver disease (NAFLD) affects some 10–30% of the general adult population worldwide^1^,^2^, with clinical and histological symptoms ranging from simple steatosis without inflammation in hepatocytes to nonalcoholic steatohepatitis (NASH) with inflammation and fibrosis^1^. To date, numerous factors have been implicated into the pathogenesis of NAFLD, and of these altered cholesterol homeostasis, including elevated cholesterol synthesis and uptake as well as reduced cholesterol excretion, has been both clinically and experimentally associated with the pathogenesis of NAFLD^3^. Moreover, further studies found that hepatic free cholesterol (FC) accumulation impaired mainly mitochondria and endoplasmic reticulum (ER) function to promote liver inflammation and fibrogenesis^4^,^5^,^6^, while conversely reduction of hepatic FC by ezetimibe improved the severity in NASH^7^,^8^,^9^,^10^.

Though the dysregulation of hepatic cholesterol metabolism has been well studied^3^, the mechanisms underlying this dysfunction remain far less clear. Characterizing these mechanisms through clinical studies remains difficult at best; in humans, the risk factors and the correlated outputs of hepatic histopathology and pathophysiology among human NAFLD are numerous and complex^11^. Fortunately, the generation of several different animal models of NAFLD has allowed for exploring both the pathogenesis and potential treatment options of human NAFLD more easily. In both mice^12^,^13^,^14^,^15^,^16^ and rabbits^17^, researchers were successful in creating a viable model by supplying a diet supplemented with cholesterol, due to its important role in the pathogenesis of NAFLD. In these models, an atherogenic diet (1.25% cholesterol and 0.5% cholate) or with the addition of high fat was successful in forcing the progression from steatosis to inflammation and fibrosis in a time-dependent manner among mice^16^, as did a combination diet of high fat (15%) and high cholesterol (1%)^18^. Alongside these models, other models, e.g., rodents^19^,^20^, opossum^21^, Ossabaw pig^22^, have provided a great deal of interesting insight and lines of useful evidence for better understanding human NAFLD.

Despite the success in the generation and application of various animal models of NAFLD, researchers are increasingly hitting roadblock in their efforts to understand and develop treatment options for human NAFLD because the myriad models continue to display distinct aspects of hepatic histopathology and pathophysiology^19^,^20^. Due to its closer evolutionary relationship to primates than more commonly used rodent models^23^, tree shrew (*Tupaia belangeri chinensis*) may be an alternative choice though it has never before been used as a model of NAFLD. More importantly, several previously studies found that tree shrews were an effective model for hepatitis B virus (HBV) and C virus (HCV) infections^24^,^25^,^26^, which itself often causes steatosis, fibrosis, and eventually hepatocellular carcinoma (HCC). Given the successes of previous studies in generating other models using a combination diet of fat, cholesterol, and cholate to partially replicate the histological features of human NAFLD in animals, it seems feasible that a similar approach could be used to develop a tree shrew NAFLD model.

In the present study, we aimed to experimentally investigate the viability of using the tree shrew as an animal model of NAFLD to explore the underlying mechanism of cholesterol by combination diet of high fat, high cholesterol, and cholate. Different groups of tree shrew were fed different diets for several weeks. Liver histopathology showed that HFHC successfully induced NAFLD from liver steatosis to inflammation and fibrosis within 10 weeks. Strikingly, the HFHC diet induced the transcriptional expression of lipoprotein lipase (*lpl*) in the liver. Furthermore, inhibition of LPL by P-407 limited the symptoms of NAFLD induced via the HFHC diet.

## Results

### HFHC caused blood dyslipidemia

Among all three groups of tree shrews, the baseline of body weight (Figure S1A), food intake (Figure S1B), and serum biochemical parameters (Table 1) were similar. Over the course of the experiment, neither diets of HFLC (high fat, low cholesterol diet) nor HFHC changed the level of fast blood glucose (FBG), but HFHC decreased the level of hemoglobin A1c (HbA1c) at 10 weeks (Table 1). Compared to the oral glucose tolerance test (OGTT) of all groups at baseline (Figure S1C), the HFLC shifted the peak to 40 minutes at 3 weeks (Figure S1D), and to 60 minutes at both 6 and 10 weeks (Figure S1E,F), indicating that HFLC caused impaired OGTT. Conversely, the HFHC diet did not exhibit any impairment of OGTT at 3 and 6 weeks, though glucose levels among this group showed a significant difference at 120 minutes (Figure S1D,E).

Compared to the control diet, both the HFLC and HFHC diets caused tree shrew dyslipidemia (Table 1). In the HFLC group, the levels of serum TC, HDL-c and LDL-c were elevated (~2 fold) at 3 weeks as compared to baseline, after week 3 the levels remained unchanged (Table 1). Administration of the HFHC diet gradually increased the levels of TC and LDL-c in a time-dependent manner from 3 to 10 weeks (Table 1). While the HDL-c level in the HFHC group was elevated at 3 weeks as compared to the baseline, but it gradually reduced as time went on (Table 1). Curiously though, the level of TG was decreased in the HFHC group, but not in HFLC group (Table 1). Additionally, the levels of AST and ALT, two widely used markers to indicate liver necroinflammation, exhibited a dramatically elevation in both the HFLC and HFHC groups in a time-dependent manner from 3 to 10 weeks (Table 1), indicating that both diets caused liver damage.

### Both HFLC and HFHC diets induced hepatic lipid accumulation

In both humans and animal models, lipid accumulation in the liver is a hallmark of NAFLD. In the control group, liver morphology (Figure S2A), weight (Figure S2B) and index (Figure S2C) remained unchanged through the duration of the experiment. Conversely, in both the HFLC and HFHC groups the livers were enlarged and the liver color changed to white (Figures S2), which was accompanied by a gradually increased liver weight and liver index in a time-dependent manner (Figure S2B,S2C). Generally, HFHC diet seemed to cause a greater effect than HFLC at any corresponding time point (Figure S2B,S2C).

**Figure 1:**
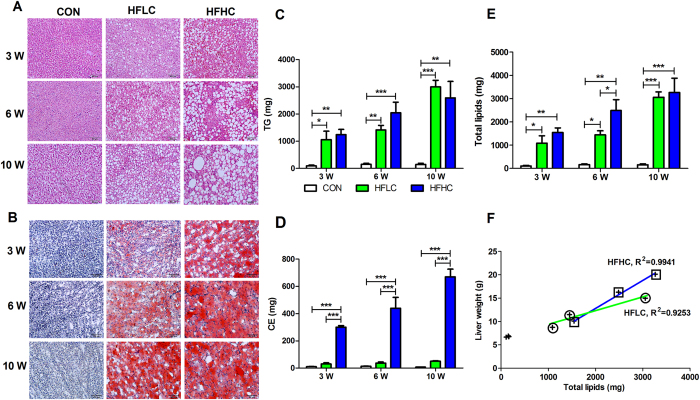
HFLC and HFHC caused lipid accumulation in liver. Liver histology showed that both HFLC and HFHC diets induced steatosis as indicated by hematoxylin-eosin (HE) staining (**A**), and Oil Red O staining (**B**). Scale bars: 100 μm. (**C**) Both HFLC and HFHC diets remarkably increased hepatic triglycerides (TG) content. (**D**) Only HFHC diet increased cholesterol esters (CE) in the liver. (**E**) Total lipids (TG + CE) in liver. (**F**) Linear relationship between liver weight and total lipids in HFHC (R^2^ = 0.9941) and HFLC (R^2^ = 0.9253). Data are presented as mean ± SEM of 3–4 animals. Significant difference between two groups, *P < 0.05, **P < 0.01, ***P < 0.001.

Liver histology via hematoxylin-eosin (HE) staining (Fig. 1A, Table 2) and Oil Red O staining (Fig. 1B) demonstrated that both HFLC and HFHC diets induced hepatic steatosis from 3 to 10 weeks, with HFHC always leading to more severe steatosis than HFLC. Tree shrews fed on both HFLC and HFHC diets eventually developed severe macrovesicular steatosis (Grade 3) at 10 weeks by the NAFLD Activity Score (NAS) (Table 2). Furthermore, analysis of hepatic lipids by thin layer chromatography/gas chromatography (TLC/GC) revealed that HFLC diet increased hepatic TG content in a time-dependent manner (Fig. 1C), slightly but not significantly increased CE content as compared to the control (Fig. 1D). Surprisingly though, HFHC diet remarkably not only increased TG content (Fig. 1C), but also CE content in a time-dependent manner from 299.53 ± 13.35 mg at 3 weeks to 670.28 ± 56.86 mg at 10 weeks (Fig. 1D). Nevertheless, the total lipids (TG + CE) in both experimental groups were nearly identical at 10 weeks (Fig. 1E). The increased liver weight in both HFLC (R^2^ = 0.9253) and HFHC (R^2^ = 0.9941) groups was positively associated with lipids accumulation in liver (Fig. 1F), suggesting that the increased liver weight primarily resulted from lipid accumulation. Collectively, these results suggest that administration of both HFLC and HFHC diets led to hepatic lipid accumulation in tree shrews.

### HFHC caused liver inflammation

**Figure 2:**
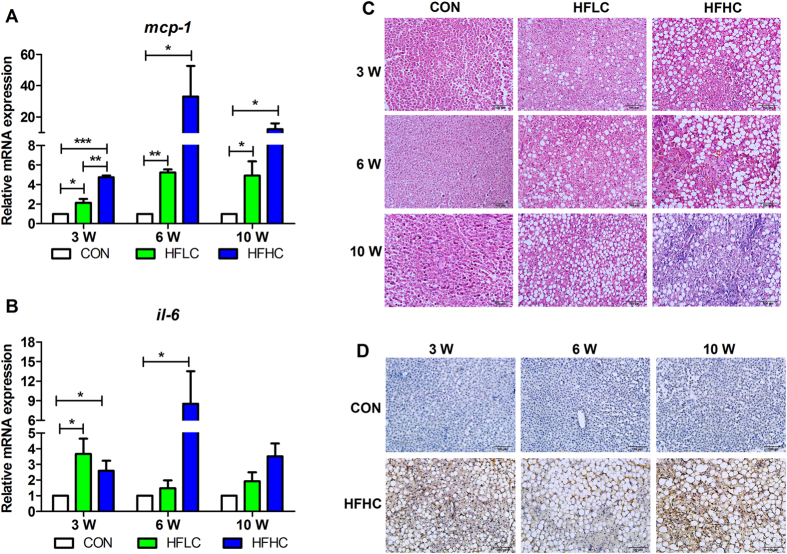
HFHC caused liver inflammation. Relative mRNA expression of *mcp-1* (**A**) and *il-6* (**B**). Data are presented as mean ± SEM of 3–4 animals. Significant difference between two groups, *P < 0.05, **P < 0.01, ***P < 0.001. C: Liver histology via hematoxylin-eosin (HE) staining showed steatohepatitis indicated by inflammatory cell clusters obviously present in HFHC group at 10 weeks. D: Immunohistochemical staining of anti-F4/80 antibody. Scale bars: 100 μm.

Cholesterol has been previously implicated in the liver inflammation in steatohepatitis in other animal models^3^. Both monocyte chemoattractant protein 1 (MCP-1) and interleukin 6 (IL-6) are often used as indicators of inflammation, and examination of the mRNA expression of liver *mcp-1* and *il-6* in both HFLC and HFHC groups was significantly increased as compared to the controls at different time point, with elevated mRNA expression of both genes in HFHC group at a generally higher level than among the HFLC group (Fig. 2A,B). This result indicates that both diets resulted in liver inflammation in tree shrews. Liver histology also displayed that steatohepatitis indicated by inflammatory cell clusters obviously present in HFHC group at 10 weeks (Fig. 2C). HFLC diet only caused mild inflammatory infiltrate (Grade 1) through the duration of the experiment, but HFHC diet caused lobular inflammation from grade 1 to 3 from 3 to 10 weeks (Table 2). Furthermore, F4/80 protein is involved in macrophage infiltration and has been widely used as a macrophage marker. Consistently, immunohistochemical staining of anti-F4/80 antibody showed that inflammation was present in the liver of HFHC diet induced tree shrews at all three stages (Fig. 2D).

### HFHC activated the expression of fibrotic genes and caused liver fibrosis

**Figure 3:**
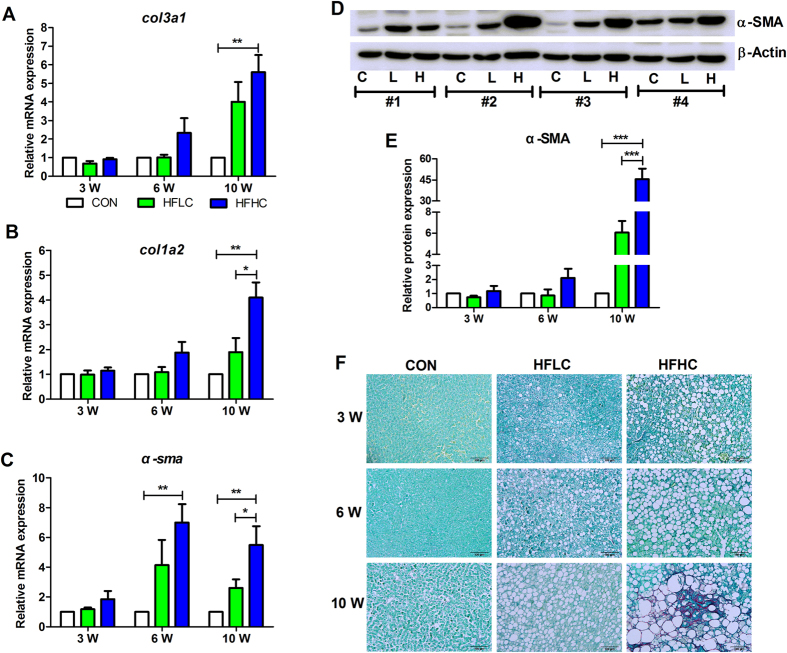
HFHC caused liver fibrosis. Relative mRNA expression of *col3a1* (**A**)*, col1a2* (**B**)*, α-sma* (**C**), encoding type III collagen, type I collagen, and alpha-smooth muscle actin, respectively. (**D**) Western blotting of α-SMA protein (10 weeks). Numbers 1–4 were four individual animals. (**E**) Relative expression of α-SMA protein to β-ACTIN. Data are presented as mean ± SEM of 3–4 animals. Significant difference between two groups, *P < 0.05, **P < 0.01, ***P < 0.001. **(F**) Sirius Red staining showed hepatic fibrosis in the HFHC group at 10 weeks. Scale bars: 100 μm.

*col3a1* and *col1a2* respectively encode a kind of type III and type I collagen, while *α-sma* encodes alpha-smooth muscle actin, all of which are commonly used as markers of fibrosis. At 3 weeks, the mRNA expression of *col3a1, col1a2* and *α-sma* remained unchanged in the liver tissues of the control, HFLC, and HFHC groups (Fig. 3A–C). By 6 weeks, the mRNA expression of *α-sma* in the HFHC group was apparently increased as compared to control (Fig. 3C), and by 10 weeks the mRNA expression of all three genes was markedly increased in the HFHC group (Fig. 3A–C). Additionally, the protein level of α-SMA increased ~45 fold in HFHC compared to control (Fig. 3D,E). Consistently, hepatic histology via Sirius Red staining showed that the HFHC diet caused hepatic perisinusoidal fibrosis (Grade 1a) at 10 weeks (Fig. 3F, Table 2), although neither the control nor HFLC diet caused fibrosis at any time point (Fig. 3F, Table 2). Together, these results indicate that HFHC diet caused liver fibrosis in tree shrews at 10 weeks.

### HFHC activated the transcriptional expression of lipoprotein lipase

**Figure 4:**
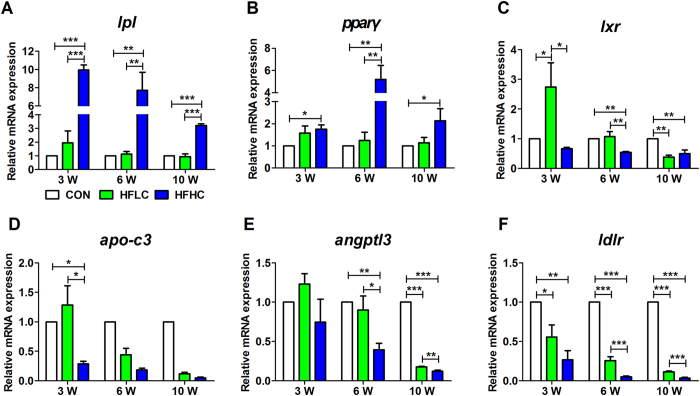
HFHC induced the expression of *lpl* and *pparγ*. QPCR revealed that HFHC induced mRNA expression of *lpl* (**A**) and *pparγ* (**B**), but reversely reduced mRNA expression of *lxr* (**C**). The relative mRNA expression of *apo-c3* (**D**), *angptl3* (E), *ldlr* (**F**). Data are presented as mean ± SEM of 3–4 animals. Significant difference between two groups, *P < 0.05, **P < 0.01, ***P < 0.001.

Dysregulation of lipid and lipoprotein metabolism plays crucial roles for NAFLD pathogenesis^27^. The expression of most genes involved in triglycerides and cholesterol metabolism, lipid oxidation were down-regulated in both HFLC and HFHC groups (Figure S3). However, the expression of *lpl* and *pparγ* was up-regulated (Fig. 4A,B). LPL is an enzyme involved in the metabolism of triglyceride-rich lipoproteins (chylomicrons and VLDL)^28^,^29^,^30^. Numerous studies have established that lipoprotein lipase (LPL) plays a crucial role in lipoprotein metabolism and transport^28^,^29^, where in the expression and activity of LPL are associated with the levels of serum TG and LDL. A decreased level of plasma TG and increased levels of plasma HDL-c and LDL-c in the HFHC group (Table 1) may potentially indicate some previously uncharacterized function of LPL in liver. The relative mRNA expression of *lpl* did not change in either the HFLC or control group over the course of the experimental period (Fig. 4A), but notably, the mRNA expression of *lpl* was increased almost 10 fold in the HFHC group at 3 weeks, and was continuously higher at 6 and 10 weeks than the level in the control group (Fig. 4A). The promoter region of *lpl* contains various elements for binding by transcription factors like peroxisome proliferator-activated receptor (PPAR)^31^ and liver X receptor (LXR)^32^. Consistently, the mRNA expression of *pparγ* (Fig. 4B), not *lxr* (Fig. 4C), was also concurrently increased with *lpl* expression only in the HFHC group, suggesting that the induction of *lpl* transcriptional expression might as a result of increased expression of *ppary*.

Meanwhile, the function of LPL requires several cofactors, such as apolipoprotein C3 (APO-C3), angiopoietin-like proteins ANGPTL3; here, the mRNA expressions of *apo-c3* and *angptl3* were concurrently decreased in a time-dependent manner in both the HFLC and HFHC groups (Fig. 4D,E). Cholesterol and cholesterol rich lipoprotein LDL may also be transported into cell through the LDL receptor (LDLR); here, the mRNA expression of *ldlr* was dramatically lower in both HFLC and HFHC groups than in control group (Fig. 4F). On the whole, these results suggest that HFHC likely activated the transcriptional expression of *lpl* via induction of *pparγ* expression and reversely down-regulated *apo-c3* and *angptl3* expression in tree shrew liver.

### Inhibition of LPL by P-407 improved the severity of liver steatosis

**Figure 5:**
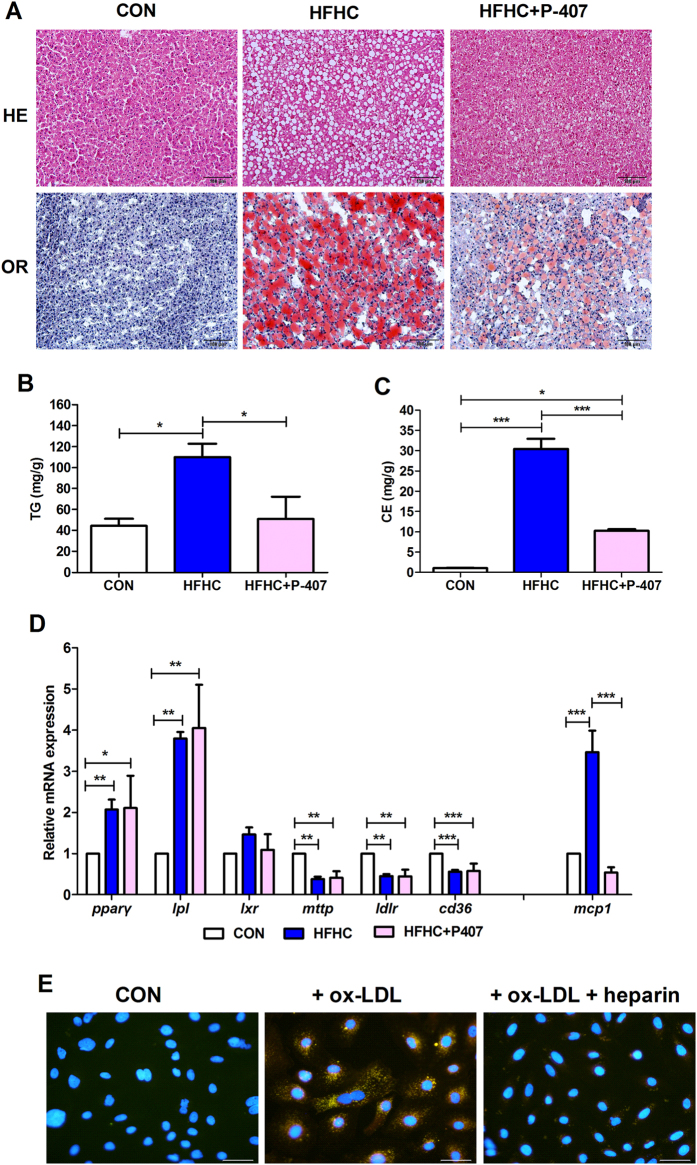
Inhibition of LPL by P-407 improved hepatic steatosis and inflammation. (**A**) Liver histology of P-407 treatment showed reduced lipid accumulation indicated by hematoxylin-eosin staining (HE, top panel) and Oil Red O (OR, bottom panel). Scale bars: 100 μm. (**B**) P-407 treatment significantly reduced TG and CE content (**C**). (**D**) Relative mRNA expression of *pparγ, lpl, lxr, mttp, ldlr* and *mcp-1*. Data are presented as mean ± SEM of 3–4 animals. Significant difference between two groups, *P < 0.05, **P < 0.01, ***P < 0.001. (**E**) Treatment of tree shrew primary hepatocytes with ox-LDL (20 μg/ml) and heparin (10 U/ml). All photos were taken with 400X magnitude under fluorescence microscope. Scale bars: 50 μm.

Since HFHC is capable of activating *lpl* expression in the liver, we hypothesized that *lpl* overexpression may be a key factor in inducing NAFLD. Previously, Poloxamer 407 (P-407) was reported to inhibit lipoprotein lipase^33^,^34^, and consequently to reduce TG accumulation in mice heart^35^. Here, liver histology showed that P-407 treatment improved hepatic steatosis with reduced lipid accumulation stained by hematoxylin-eosin (HE) and Oil Red O (OR) (Fig. 5A), as well as quantified TG and CE content by TLC/GC (Fig. 5B,C). Consistently, treatment of tree shrew primary hepatocytes with heparin, which releases LPL from its tissue binding sites, observably reduced lipid accumulation induced by ox-LDL (Fig. 5E). Meanwhile, the mRNA expression of *pparγ, lpl, lxr, mttp, ldlr* and *cd36* was not affected by P-407, but the expression of liver inflammation marker *mcp-1* (Fig. 5D) as well as the level of serum AST and ALT (Table S1) were dramatically reduced, suggesting improvement of steatosis and inflammation. However, whole body inhibition of LPL definitely exaggerated the increased levels of TC, TG, LDL-c, HDL-c, FBG in HFHC group (Table S1), since that might impair the normal function of LPL in lipid utilized tissues like heart, muscle, etc.

## Discussion

Diet is a well-recognized risk factor of NAFLD among humans. High-fat and cholesterol diets with cholate^16^ or without cholate^18^ have been previously found to induce mice NAFLD that progressed from liver steatosis to inflammation and fibrosis. Extending this approach of using HFHC diet to tree shrew yielded a similar results, though at a much brisker pace (10 weeks, Figs 1, 2, 3) than previously seem among rodents (24–30 weeks)^16^,^18^, suggesting that tree shrew maybe a more easily generated animal model of human NAFLD.

Various diets have been found to lead to distinctive hepatic histopathology and pathophysiology in both humans and experimental animals, even in different rodent species and strains^19^,^36^. Human NAFLD is often accompanied with metabolic abnormalities such as obesity, dyslipidemia^11^,^37^,^38^,^39^, but neither the HFLC nor HFHC diet was shown to change the body weight in tree shrews over the course of this 10 week experiment (Figure S1A). Part of this result may be explainable by variance previously observed across different models. Here, the HFHC diet was similar to atherogenic diet used by Matsuzawa *et al.*^16^ which resulted in a 9% body weight loss in mice after 24 weeks, but in Ossabaw pigs a similar diet (46% fat and 2% cholesterol and 0.7% cholate) led to an increase in body weight^22^, as did a diet of 15% fat and 1% cholesterol without cholate in mice after 30 weeks^18^. Although the majority of NAFLD patients are obese, a high percentage (15–21%) of Asia-Pacific NAFLD subjects have been found to be non-obese^40^,^41^. The tree shrew models of NAFLD induced by both HFLC and HFHC diets may be useful for future research of non-obese NAFLD.

In humans, rodents and rabbits, diets containing cholesterol often lead to increased levels of plasma cholesterol. Here, both HFLC and HFHC diets consistently significantly increased the level of plasma cholesterol in a time- and cholesterol-dependent manner (Table 1), but in a previous study on mice the atherogenic diet led to a decrease of plasma HDL-c level^16^ in one instance but not in another^14^. However, our HFHC diet increased plasma HDL-c level (Table 1). Similarly, among the tested tree shrews HFHC diet dramatically increased plasma LDL-c level (Table 1), consistent with the previous reports from mice^14^,^16^, but reduced the plasma TG level (Table 1).

In previous animal models, the atherogenic diet was reported to reduce hepatic TG level^14^,^16^ in rodents, but increase the hepatic TG level in Ossabaw pigs^22^. Here, in the new tree shrew model, the HFHC diet significantly increased both hepatic TG and cholesterol level in a time-dependent manner (Fig. 1C,D). This specific discrepancy notwithstanding, all these studies, including ours, consistently found that high level cholesterol in a diet significantly increased hepatic cholesterol levels (Fig. 1D), which is consistent with results found in human NASH and NAFLD patients^42^.

It has been well established that hepatic cholesterol is mainly originated from *De Novo* synthesis and circulation in the form of LDL through internalization via receptor-mediated endocytosis by LDL receptors (LDLR) in liver^3^,^43^. However, the expression of genes involved in cholesterol synthesis (Figure S3A–H) and internalization (*ldlr)* (Fig. 4F) was dramatically repressed in HFHC group, indicating that the accumulated hepatic cholesterol might not be mediated by these pathways. Although LPL is not supposed to transcriptionally express in murine adult liver^44^, that may due to the repression by miR-467b^45^ and miR-29a^46^. Inhibition of miR-29a causes LPL-dependent hepatic lipid uptake and steatosis^46^. On the contrary, overexpression of LPL in liver leaded to liver TG accumulation^47^. Moreover, heart-specific LPL deletion mice had defective lipid accumulation in heart^35^. Here the HFHC diet led to a significant induction of mRNA expression of *lpl* in the liver of tree shrew (Fig. 4A). The HFHC diet contains cholesterol (1.25%) and cholate (0.5%), which were previously reported to regulate the expression of distinct genes involved in acute inflammation and extracellular matrix deposition in hepatic fibrosis, respectively^14^. The induced mRNA expression of *lpl* could then be due to cholesterol, not cholate, since high concentration of cholesterol (2%) alone could activate mRNA expression of *lpl* via LXR in mice^32^. Consistently, in our pre-experiment, we found an increase of LPL mRNA expression as well as a corresponding decrease of the level of plasma TG decreased in a cholesterol-dependent manner (Figure S4). Whereas, the mechanisms of cholesterol transporting into liver through LPL but not LDLR under different cholesterol diets need to be further characterized in the future.

**Figure 6:**
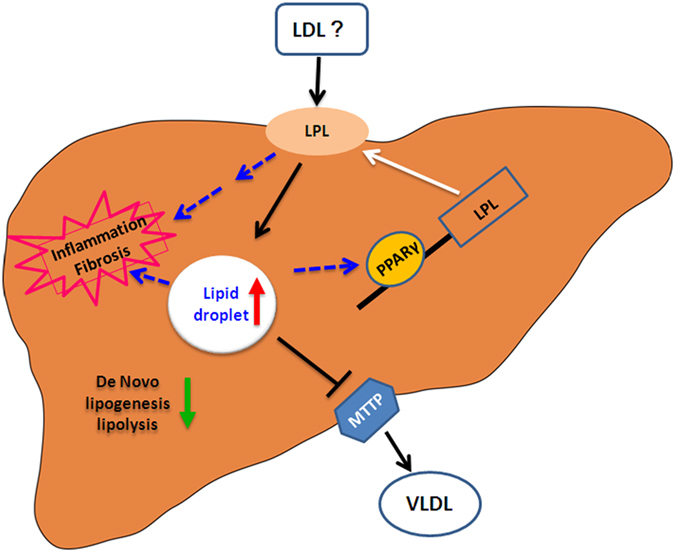
Proposed model of HFHC induced NAFLD in tree shrew. PPARγ, which maybe induced by cholesterol or unknown lipid, activated the transcription of LPL to further catalyze and transport circulation lipids (LDL-c or others) into the liver. Concurrently, *De Novo* synthesis, oxidation, and export of lipids were down-regulated, eventually leading to lipid accumulation, inflammation and fibrosis in the liver of tree shrew.

Inhibition of LPL activity by P-407 significantly prevented hepatic lipid accumulation (Fig. 5A–C) as well as inflammation indicated by the decreased expression of hepatic *mcp-1* (Fig. 5D) and serum AST and ALT levels (Table S1) in HFHC group. Additionally, heparin treatment also reduced lipid accumulation in primary hepatocytes of tree shrew induced by ox-LDL (Fig. 5E). Those results suggest that increased lipid accumulation in the HFHC group was probably a result of activated LPL in the liver (Fig. 6). Consistently, in the livers of morbidly obese patients who had higher hepatic TG and TC than healthy controls, the mRNA expression and activity of LPL was significantly higher than in the control livers, and was also correlated with the severity of the liver damage^48^. Similarly, the mRNA expression of PPARγ was also up-regulated in the liver of obese NAFLD patients with steatosis and steatohepatitis^49^. Thus, it will be very curious to clinically test whether specific inhibition of hepatic LPL may relieve the hepatic symptoms in these obese NAFLD patients.

Lipid deposition accompanying NAFLD was previously reported to be a result of impaired lipid metabolism^27^. Here, the expression of many genes involved in cholesterol metabolism, triglycerides metabolism, and fat oxidation were down-regulated in both HFLC and HFHC groups (Figure S3). Meanwhile, the expression of *mttp*, encoding microsomal TG transfer protein (MTTP) for VLDL synthesis and export from liver, was significantly decreased in the HFHC group (Figure S3H). Taken collectively, these results suggest that administration of HFHC diet impairs cholesterol and triglycerides metabolism, and reduces fat oxidation and lipid export, leading to lipid deposition in the liver in tree shrews (Fig. 6).

## Methods

### Animals and experimental design

Male tree shrews around one year of age raised at the Kunming Institute of Zoology, Chinese Academy of Science were divided into two experimental groups along with one control group. The two experimental groups were either fed a high-fat and low-cholesterol (HFLC, #D12079B, 21% fat and 0.21% cholesterol by weight) or a high-fat and high-cholesterol diet supplemented with cholate (HFHC, #D12109C, 20% fat, 1.25% cholesterol and 0.5% sodium cholate by weight), while the control group was fed a regular diet (CON, #D12102C, 4% fat by weight). Tree shrews in all three groups were fed with control diet two weeks prior to initiating the experiments. At four set time points (0, 3, 6, and 10 weeks) random animals (n = 4–8) from each group were selected for harvesting, and were fasted overnight (14 hours) prior to being euthanized by ethyl ether anesthesia. All animal experiments were carried out according to the guidelines approved by the Animal Ethics Committee of the Kunming Institute of Zoology, Chinese Academy of Science (Approval number: SYDW20120105001).

### Oral glucose tolerance test (OGTT)

OGTT was performed as previously described^50^. In pre-experiment, tree shrews fed on HFHC at 10 weeks died soon after OGTT, thus, OGTT was not applied for the HFHC group at 10 weeks.

### Blood and liver sampling

After being euthanized, blood and liver samples were harvested; liver samples were fixed in 10% formalin, or snap frozen in liquid nitrogen and stored at −80 °C for later analyses. Blood sampling and handling were performed following previously described^50^.

### Analysis of serum biochemical parameters

The plasma levels of AST, ALT, TG, TC, LDL-c, HDL-c, glucose, and hemoglobinA1c (HbA1c) in the blood were assayed by an automatic blood biochemistry analyzer (Abbott CI16200, Chicago, USA) at the First People’s Hospital of Yunnan Province, China (Kunming, China).

### Hepatic histology and immunohistochemical staining of anti-F4/80 antibody

From the liver samples, 5 μm-thick sections of formalin-fixed and paraffin-embedded liver tissues were processed for hematoxylin-eosin (HE) staining, Sirius Red and fast green FCF staining^51^. For Oil Red O staining, liver tissues were first embedded in an OCT compound and then 30-μm-thick sections were cut and further stained with Oil red O to identify neutral lipids (red). Nuclei were counter-stained (blue) with hematoxylin.

The performance of immunohistochemistry was followed as the described^52^, but with minor modification, in which the hepatic sections were developed with a DAB kit (Fuzhou Maixin Biotech. Co., Ltd., Fuzhou, China) and the nuclei were stained by hematoxylin.

### Analysis of hepatic lipids

Total lipids of liver tissues were extracted according to Folch^53^. To separate lipid species, 30 μl total lipids were loaded to one-dimensional thin-layer chromatography (TLC), using hexane-diethyl ether-glacial acetic acid (75:35:1, v/v/v) as solvent^54^. Analysis of resulting lipids was done by gas chromatography (GC) as reported in one of our previous studies^55^. Lipids were quantified with C15:0 as an internal standard.

### Real-time quantitative PCR of hepatic tissue

Total RNA was extracted from liver tissues using *TransZol* reagent (TransGen Biotech, Beijing, China), and reverse transcribed to complementary DNA using a PrimeScript RT reagent Kit (TaKaRa Biotechnology, Dalian, China). Real-time quantitative PCR was performed on a ABI 7900HT sequencer (Applied Biosystems, Foster City, USA). *β-actin* was used as a standard control to normalize the relative mRNA expression of a specific gene via the ΔΔCt method. Sequences of all primers used in this study are available upon request.

### Western blotting

Liver tissues were ground and homogenized at 4 °C with an extraction buffer. The tissue homogenates were then centrifuged, and the supernatants were used for western blotting analysis, with α-smooth muscle actin (Abcam, Hong Kong, China) and β-actin (Sigma-Aldrich, St. Louis, USA) as the primary antibodies and Goat Anti-Rabbit IgG, HRP (Thermo-Fisher Scientific, Rockford, USA) and Goat Anti-Mouse IgG, HRP (Thermo-Fisher Scientific, Rockford, USA) as the secondary antibodies.

### Poloxamer 407 treatment

A solution of Poloxamer 407 (P-407) (Sigma-Aldrich, St. Louis, USA) was prepared with normal saline at a final concentration at 200 mg/ml. The solution was incubated at 4 °C overnight to facilitate dissolution of P-407 as previously reported^56^. Tree shrews were administered with P-407 solution at the dosage of 1 g/kg body weight by intraperitoneal (i.p.) injection once every two days for 3 weeks.

### ox-LDL and heparin treatment to tree shrew primary hepatocytes

Tree shrew primary hepatocytes were cultured with Dulbecco’s Modified Eagle Medium (Life Technologies, Grand Island, USA) supplemented with 10% fetal bovine serum (Life Technologies, Grand Island, USA) as well as 100 U/ml penicillin and streptomycin in 5% CO_2_ at 37 °C. The medium was changed once every 3 days. Tree shrew primary hepatocytes were first grown on coverslips for 2 days, and then pre-incubated in serum free medium for 4 hours, after that, 20 μg/ml ox-LDL (Luwen Biotechnologies, Shanghai, China) and 10 U/ml heparin sodium were subsequently added. After 20 hours, cells were fixed by 4% paraformaldehyde in sodium phosphate for 1 hour at room temperature. And then, coverslips were washed with ddH_2_O for 10 minutes, stained with 17 ng/ml Nile Red (Eugene, Oregon, USA) for 10 minutes, second ddH_2_O washed for 10 minutes, counterstained with DAPI (Sigma-Aldrich, St. Louis, USA) for 2 minutes, third ddH_2_O washed for 10 minutes, mounted on glass slides with Glycerol Jelly Mounting Medium (Beyotime, Jiangsu, China) to visualize lipid droplets under fluorescence microscope (Olympus, BX53, Japan).

### Statistical analysis

Data are presented as mean ± SEM, except when specifically indicated. Statistical analysis performed included t-test or analysis of variance (ANOVA) followed by LSD multiple comparisons using SPSS20.0 (IBM SPSS Statistics, Armonk, NY, USA). *P* < 0.05 was considered statistically significant. All figures were made using GraphPad Prism 5 (GraphPad Software, La Jolla, CA, USA).

## Additional Information

**How to cite this article**: Zhang, L. *et al.* Cholesterol induces lipoprotein lipase expression in a tree shrew (*Tupaia belangeri chinensis*) model of non-alcoholic fatty liver disease. *Sci. Rep.* **5**, 15970; doi: 10.1038/srep15970 (2015).

## Supplementary Material

Supplementary Information

## Figures and Tables

**Table 1:** Blood biochemical parameters.

	CON				HFLC				HFHC			
	0 W (n = 8)	3 W (n = 8)	6 W (n = 8)	10 W (n = 8)	0 W (n = 8)	3 W (n = 8)	6 W (n = 8)	10 W (n = 5)	0 W (n = 8)	3 W (n = 8)	6 W (n = 6)	10 W (n = 4)
AST(U/L)	194.8 ± 28.4	161.5 ± 15.6	192.6 ± 25.0	173.1 ± 19.6	174.3 ± 21.5	236.1 ± 33.9	573.5 ± 139.7b^†^ d*	1097.2 ± 194.6c^†^ e^†^ f^†^	211.4 ± 36.8	397.4 ± 30.9 a^†^	1754.8 ± 142.5 b^†^ d^†^	3844.0 ± 653.5 c^†^ e^†^ f^†^
ALT(U/L)	162.0 ± 46.6	151.6 ± 20.6	160.6 ± 37.0	121.3 ± 16.7	93.4 ± 11.9	285.8 ± 56.2 a^†^	636.8 ± 121.8 b^†^ d^†^	961.0 ± 126.0 c^†^ e^†^ f*	127.5 ± 34.4	333.4 ± 27.9 a^†^	1728.8 ± 228.4 b^†^ d^†^	3111.5 ± 667.7 c^†^ e^†^ f^†^
TC(mmol/L)	1.65 ± 0.08	1.68 ± 0.09	1.99 ± 0.09	1.91 ± 0.10	1.63 ± 0.10	4.05 ± 0.45 a^†^	3.89 ± 0.33 b^†^	4.18 ± 0.25 c^†^	1.66 ± 0.10	6.13 ± 0.73 a^†^	10.09 ± 1.51 b^†^ d*	13.16 ± 2.27 c^†^ e^†^
TG(mmol/L)	0.34 ± 0.03	0.28 ± 0.03	0.31 ± 0.04	0.25 ± 0.03	0.34 ± 0.04	0.27 ± 0.03	0.29 ± 0.05	0.23 ± 0.03	0.30 ± 0.03	0.06 ± 0.01 a^†^	0.06 ± 0.01 b^†^	0.08 ± 0.03 c^†^
HDL-c(mmol/L)	1.16 ± 0.08	1.30 ± 0.07	1.26 ± 0.08	1.33 ± 0.08	1.15 ± 0.06	2.15 ± 0.15 a^†^	1.99 ± 0.13 b^†^	2.10 ± 0.08 c^†^	1.28 ± 0.03	3.05 ± 0.19 a^†^	2.70 ± 0.25 b^†^	1.64 ± 0.11 e^†^ f^†^
LDL-c(mmol/L)	0.31 ± 0.02	0.29 ± 0.04	0.38 ± 0.04	0.31 ± 0.01	0.29 ± 0.03	0.58 ± 0.06 a^†^	0.54 ± 0.09 b*	0.43 ± 0.09	0.34 ± 0.03	0.98 ± 0.08 a*	2.67 ± 0.41 b^†^	4.86 ± 0.79 c^†^ e^†^ f^†^
FBG(mmol/L)	4.29 ± 0.25	4.20 ± 0.20	4.82 ± 1.14	3.45 ± 0.30	3.93 ± 0.34	4.70 ± 0.20	4.14 ± 0.23	4.12 ± 0.36	4.43 ± 0.23	4.06 ± 0.34	4.17 ± 0.55	3.90 ± 0.79
HbA1c(%)	4.04 ± 0.21	3.81 ± 0.14	4.11 ± 0.11	3.65 ± 0.20	4.12 ± 0.15	3.67 ± 0.28	3.25 ± 0.36 b*	4.08 ± 0.09	4.07 ± 0.15	4.38 ± 0.05	4.16 ± 0.12	2.02 ± 0.75 c^†^ e^†^ f^†^

Data are presented as mean ± SEM. a, b, c, d, e, f: indicated a significant difference of comparison in same group at different time point (*P  <  0.05, ^†^P  <  0.01). a: 0 W versus 3 W, b: 0 W versus 6 W, c: 0 W versus 10 W, d: 3 W versus 6 W, e: 3 W versus 10 W, f: 6 W versus 10 W.

**Table 2:** NAS Score.

	3 weeks			6 weeks			10 weeks		
	CON	HFLC	HFHC	CON	HFLC	HFHC	CON	HFLC	HFHC
Steatosis (0–3)	0	1	2	0	2	3	0	3	3
Lobular inflammation (0–3)	0	1	1	0	1	2	0	1	3
Fibrosis (0–4)	0	0	0	0	0	0	0	0	1a

The standards for NAS Score was followed by Savard *et al*.^18^.

Steatosis is graded as: < 5% (0); 5%–33% (1); 34%–66% (2); and >66% (3).

Lobular Inflammation combines mononuclear, fat granulomas, and polymorphonuclear leucocytes and is graded as: none (0); < 2 per 200 magnification (1); 2–4 per 200 magnification (2); and > 4 per 200 magnification (3).

Fibrosis is staged as: none (0); perisinusoidal (1a); periportal (1b); periportal and perisinusoidal (2); bridging fibrosis (3); and cirrhosis (4).
