# Supplementary material for: Cholesterol induces lipoprotein lipase expression in a tree shrew (Tupaia belangeri chinensis) model of non-alcoholic fatty liver disease
Source: Sci Rep. 2015 Nov 2;5:15970. doi: 10.1038/srep15970 (PMC4629153; doi:10.1038/srep15970)
Supplement: Supplementary Information [file srep15970-s1.pdf]

Cholesterol induces lipoprotein lipase expression in a tree shrew (*Tupaia belangeri chinensis*) model of non-alcoholic fatty liver disease

Linqiang Zhang [1,2*], Zhiguo Zhang[1,2*],Yunhai Li[1*],Shasha Liao[1,3], Xiaoyun Wu[1], Qing Chang[1], Bin Liang[1#]

1,Key Laboratory of Animal Models and Human Disease Mechanisms of Chinese Academy of Science & Yunnan province, Kunming Institute of Zoology, Chinese Academy of Sciences , Kunming, Yunnan 650223, China

2, Kunming College of Life Science, University of Chinese Academyof Sciences, Kunming, Yunnan 650204, China

3, School of Life Sciences, Anhui University, Hefei, Anhui 230601, China.

*: Equal contribution

#: Correspondence should be addressed to

Bin Liang

Tel: 086-871-65181927

E-mail: liangb@mail.kiz.ac.cn

Supplementary figures and legends

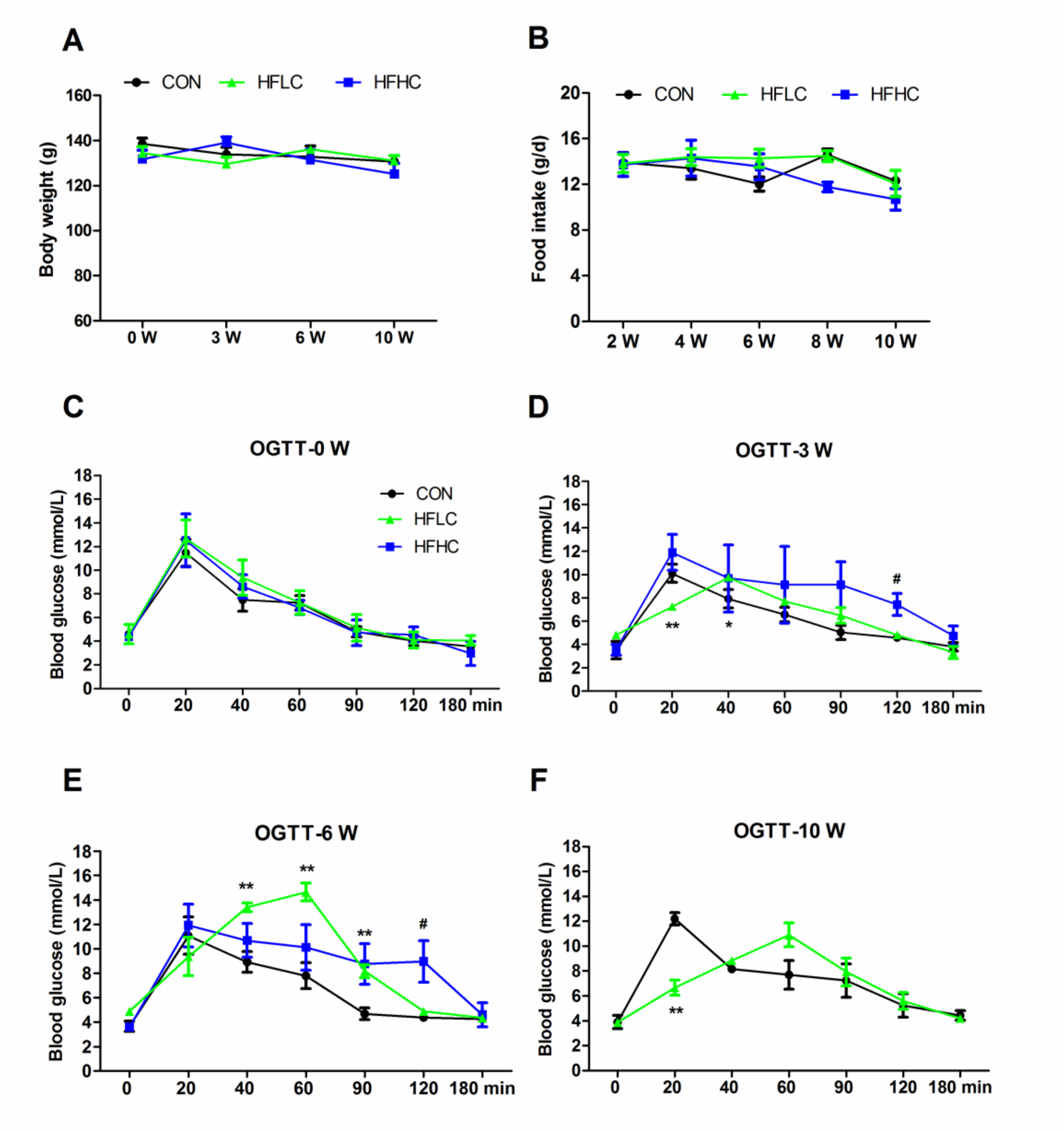

Figure S1. Body weight, food intake and oral glucose tolerance test (OGTT) in tree shrew. Compared to control diet, both HFLC and HFHC diet did not change body weight (A) and food intake (B). OGTT at 0 week (C) (n=4 for each group), 3 weeks (D) (n=4, 4, 5 for CON, HFLC and HFHC, respectively), 6 weeks (E) (n=4, 4, 3 for CON, HFLC and HFHC, respectively), and 10 week (F) (n=3, 3 for CON and HFLC, respectively). Data were presented as mean ± SEM. Significant difference between two groups, *: $P<0.05$, **: $P<0.01$.

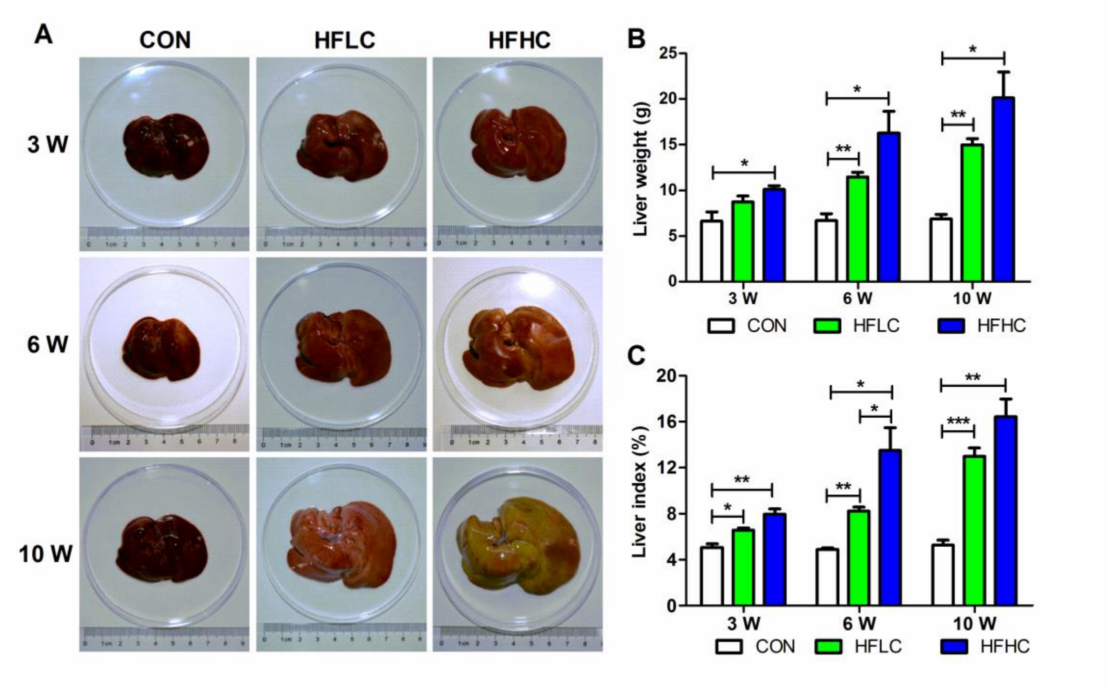

Figure S2. Liver morphology, liver weight and liver index. A: liver morphology of represented animal. Liver weight (B) and liver index (C) were significantly increased in a time-dependent manner in both HFLC and HFHC group compared to control group (CON). Data were presented as mean ± SEM of 3-4animals. Significant difference between two groups, *: $P<0.05$, **: $P<0.01$, ***: $P<0.001$.

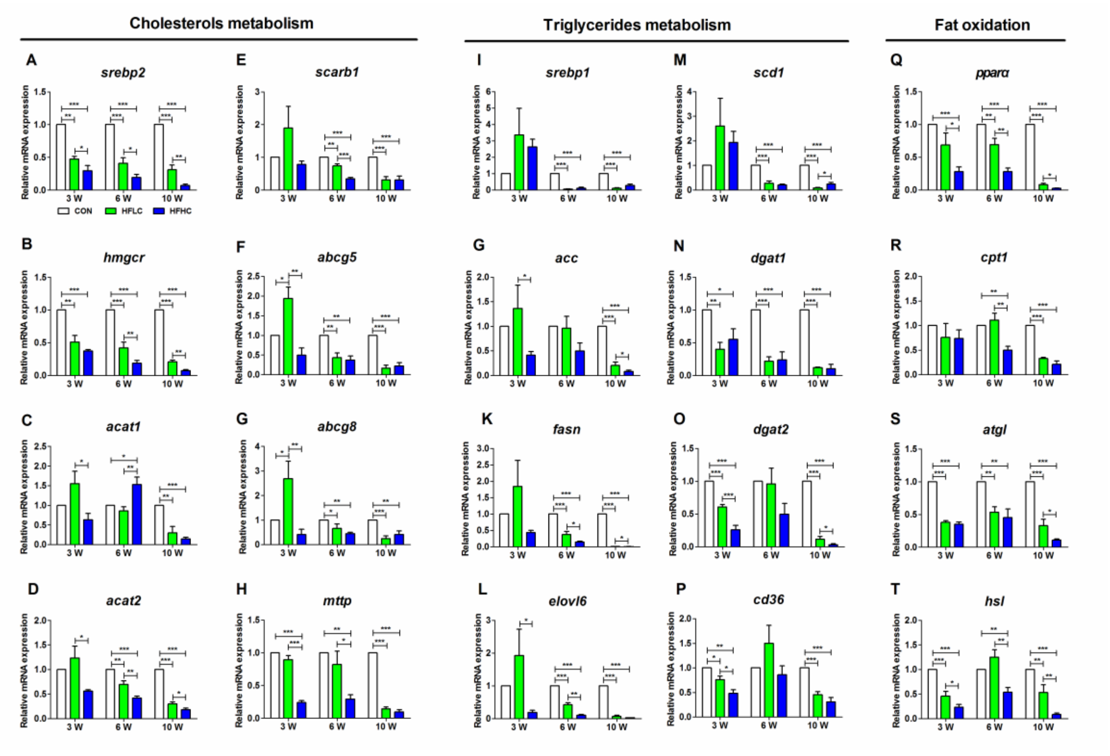

Figure S3.The mRNA expression of genes involved in cholesterol, triglycerides metabolism and fat oxidation. A-H: Relative mRNA expression of *srebp2*, *hmgcr*, *acat1*, *acat2*, *scarb1*, *abcg5*, *abcg8* and*mttp* involved in cholesterol metabolism. I-P: Relative mRNA expression of *srebp1*, *acc*, *fas*, *elovl6*, *scd1*, *dgat1*, *dgat2* and *cd36* involved in triglycerides metabolism. Q-T: Relative mRNA expression of *pparα*, *cpt1*, *atgl* and *hsl* involved in fat oxidation. Data were presented as mean ± SEM of 3-4animals. Significant difference between two groups, *: P<0.05, **: P<0.01, ***: P<0.001. **Abbreviations:** SREBP: Sterol regulatory element binding protein; HMGCR: 3-hydroxy-3-methyl-glutaryl-CoA reductase; ACAT: Acyl-coenzyme A cholesterol acyltransferase; SCARB: Scavenger receptor class B member; ABCG: ATP-binding cassette sub-family G member; MTTP: Microsomal triglyceride transfer protein; ACC: Acetyl-CoA carboxylase; FASN: Fatty acid synthase; ELOVL6: Family member 6, elongation of long-chainfatty acids; SCD: Stearoyl-CoA desaturase; DGAT: Diacylglycerol acyltransferase; CD-36: Cluster differentiation protein-36, fatty acid translocase; CPT-1: Carnitine palmitoyltransferase-1; ATGL: Adipose triacylglycerol lipase; HSL: Hormone-sensitive lipase.

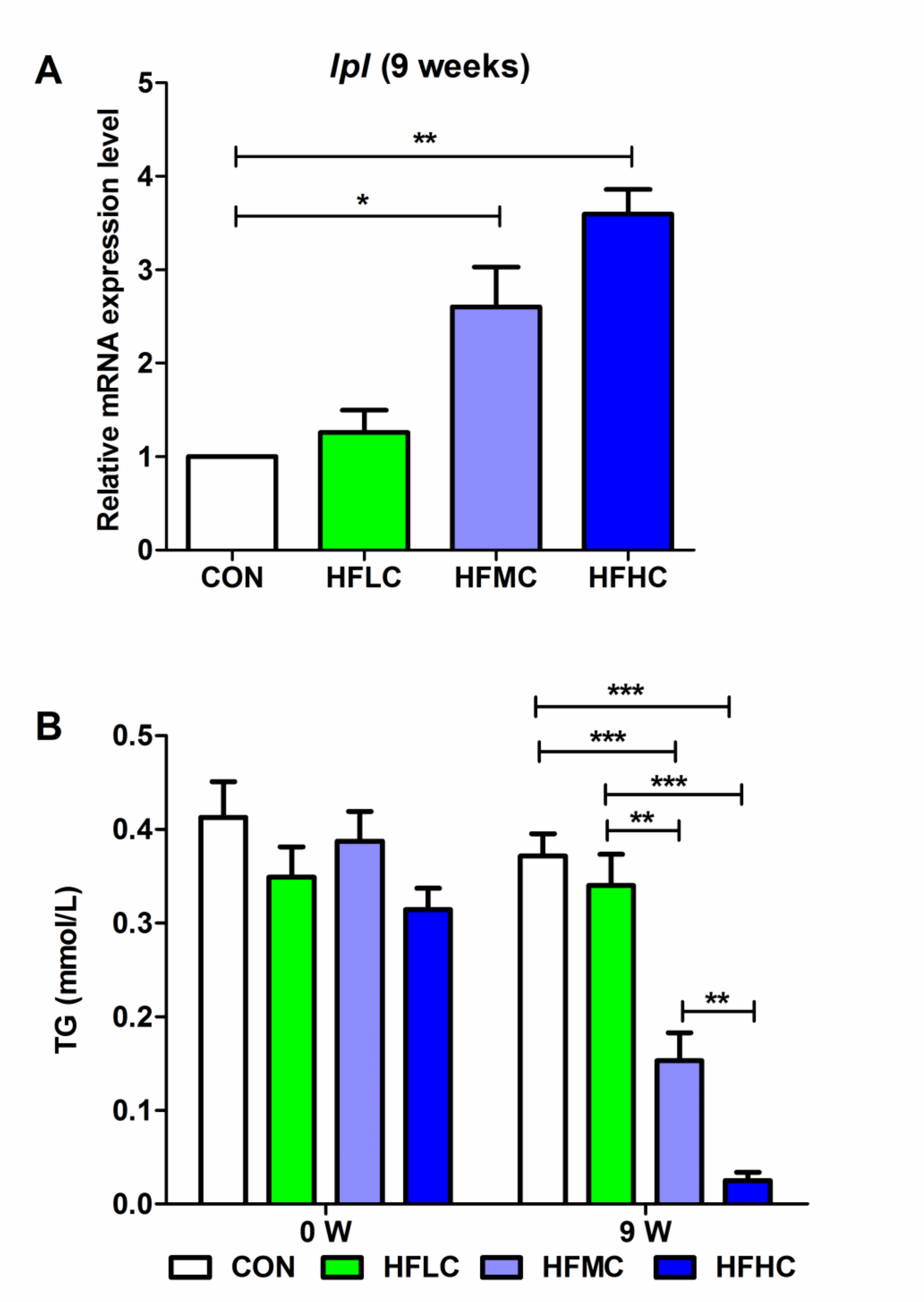

Figure S4. The mRNA expression of *lpl* and plasma TG level. A: The mRNA expression of hepatic *lpl* among different diet groups at 9 weeks. HFMC: high fat (20.2%), medium cholesterol (0.5%) diet (#D12107C). B: Plasma TG level in different diet groups at 9 weeks. Data were presented as mean ± SEM of 3-4 animals for hepatic *lpl*, 6-8 animals for plasma TG, respectively. Significant difference between two groups, **: P<0.01, ***: P<0.001.

Supplementary table

	CON		HFHC		HFHC+P-407	
	0 W (n=6)	3 W (n=6)	0 W (n=5)	3 W (n=5)	0 W (n=6)	3 W (n=3)
Body weight (g)	136.0±2.6	137.8±2.7	131.0±3.1	123.9±6.0	130.0±5.1	120.2±7.4 b*
AST(U/L)	177.8±45.6	133.0±19.4	113.2±35.5	304.6±42.6 a† d†	145.7±32.3	55.3±43.8 c†
ALT(U/L)	127.8±25.8	109.8±17.1	140.6±70.3	409.4±84.8 a† d*	124.7±38.4	45.33±2.4 c†
TC(mmol/L)	2.03±0.14	1.71±0.09	2.03±0.09	7.80±0.51 a† d†	2.01±0.17	31.15±0.47 b† c† d†
TG(mmol/L)	0.44±0.02	0.47±0.04	0.45±0.03	0.28±0.04 a* d*	0.40±0.05	4.20±0.70 b* c* d*
HDL-c(mmol/L)	1.21±0.10	0.98±0.09	1.18±0.07	2.78±0.17 a† d†	1.23±0.10	6.48±2.82 b† c* d*
LDL-c(mmol/L)	0.22±0.05	0.28±0.03	0.17±0.02	2.29±0.43 a† d†	0.19±0.03	18.77±0.58 b† c† d†
FBG(mmol/L)	4.17±0.30	4.28±0.29	4.34±0.46	4.58±0.26	4.07±0.40	5.57±0.24 b* d*
HbA1c(%)	4.65±0.08	4.06±0.19 d*	4.58±0.06	4.54±0.07 a*	4.25±0.17 b†	3.17±0.12 b† c† d†

Table S1. Body weight, food intake, and blood biochemical parameters of tree shrew treated with P-407. Data are presented as mean±SEM. a, b, c: indicated a significant difference of comparison between groups at same time point (*P<0.05, †P<0.01). a: CON versus HFHC, b: CON versus HFHC+P-407, c: HFHC versus HFHC+P-407.d: indicated a significant difference of comparison in same diet group at different time point (*P<0.05, †P<0.01). d: 0 W versus 3 W.
